# Implementation of Trusted Traceability Query Using Blockchain and Deep Reinforcement Learning in Resource Management

**DOI:** 10.1155/2022/6559517

**Published:** 2022-09-19

**Authors:** Yunting Jiang, Yalin Lei

**Affiliations:** ^1^School of Economics and Management, China University of Geosciences, Beijing 100083, China; ^2^Key Laboratory of Carrying Capacity Assessment for Resource and Environment, Ministry of Natural Resources of the People's Republic of China, Beijing 100083, China

## Abstract

To better track the source of goods and maintain the quality of goods, the present work uses blockchain technology to establish a system for trusted traceability queries and information management. Primarily, the analysis is made on the shortcomings of the traceability system in the field of agricultural products at the present stage; the study is conducted on the application of the traceability system to blockchain technology, and a new model of agricultural product traceability system is established based on the blockchain technology. Then, a study is carried out on the task scheduling problem of resource clusters in cloud computing resource management. The present work expands the task model and uses the deep Q network algorithm in deep reinforcement learning to solve various optimization objectives preset in the task scheduling problem. Next, a resource management algorithm based on a deep Q network is proposed. Finally, the performance of the algorithm is analyzed from the aspects of parameters, structure, and task load. Experiments show that the algorithm is better than Shortest Job First (SJF), Tetris^*∗*^, Packer, and other classic task scheduling algorithms in different optimization objectives. In the traceability system test, the traceability accuracy is 99% for the constructed system in the first group of samples. In the second group, the traceability accuracy reaches 98% for the constructed system. In general, the traceability accuracy of the system proposed here is above 98% in 8 groups of experimental samples, and the traceability accuracy is close for each experimental group. The resource management approach of the traceability system constructed here provides some ideas for the application of reinforcement learning technology in the construction of traceability systems.

## 1. Introduction

Generally, source traceability can be used to measure the quality and value of goods, which is of profound significance for both merchants and consumers. For a long time, only written instructions are used as evidenced product source, such as the customs-confirmed origin certificate of imports [1, 2]. This, however, poses security risks in terms of traceability technology. With the continuous development of science and technology, Blockchain Technology (BT) is taking over the originally risky traceability technologies by presenting many advantages, including immutability, transparency, and distributed database. BT can better solve data trust problems. Thus, more researchers are using BT to query trusted traceability [3].

At present, there are the following defects in the traceability of the supply chain system. (1) The root cause of the problems of counterfeit, shoddy, and tampering of goods in the transportation and trading process of the supply chain comes from the information asymmetry among the central institutions, producers, and consumers, which leads to the problem of trust between the two sides of the transaction, which is also one of the core issues of the authenticity of goods. A commodity traceability system is a key method to solve information asymmetry. (2) Depending on a unified central database, data may be tampered with in storage, transmission, display, and other scenarios; commodity tracking system is still in a state of manual operation in many links, for information providers can selectively shield basic information adverse to themselves; commodity tracking depends on the strength of the regulatory measures of the central institution. The system has space for manual operation but no effective restriction on the rights of regulators. When an accident happens in the process of supply chain commodity transportation, although producers promise to compensate customers, the compensating process involves the traceability of information. Hence, there are some difficulties to implement the compensation. Blockchain can make up for this defect. According to the principle of blockchain, its application should realize the establishment of a trust relationship for platform users under the mode of sharing economy, so that they can supervise each other. There are six steps for applying the blockchain in the distribution of the supply chain: (1) User A sends out instructions to user B; (2) Transaction data are recorded in the “block” of the network; (3) Blocks transmit transaction data to all members of the network; (4) Members verify whether the transaction is effective; (5) Blocks are then added to the blockchain, leaving unmodifiable logistics records; (6) Transactions on the supply chain are completed. The technical characteristics of blockchain make the transaction data between users more realistic and credible. In the blockchain system, each record information generated requires multiple participants to reach a consensus, and the rights of each participant are equal. Each participant in the blockchain stores all the transaction data recorded in the whole chain. Even if a participant node is attacked or damaged, it will not have any bad impact on the blockchain.

The origin and value of product traceability problems are expounded in the first section, along with the research schema.  Section 2 is the literature review; Section 3 introduces BT and information management technology, then designs the solution scheme of trusted traceability and BT-based RM, and finally, presents the experimental parameter configuration. In Section 4, the field test is conducted on the BT to verify the performance of the trusted traceability solutions proposed from the aspects of storage and time cost. Besides, the performance of the RM scheme is also investigated from the aspects of the average slowdown and the average completion time of minimizing tasks in the task scheduling.  Section 5 summarizes the experiment and points out the expected improvement and prospects for future work.

Innovations can be divided into two aspects. In the task scheduling problem of resource clusters in cloud computing resource management, referring to the existing task model, the task model is expanded into single-stage tasks and multistage tasks. Besides, the Q Learning-Convolutional Neural Network (DNQ) algorithm in deep reinforcement learning is used to minimize the average task slowdown and complex optimization objectives such as minimizing average task completion time and maximizing task priority discrimination. On the issue of traceability system design, the present work designs the blockchain traceability schemes such as the process, data storage, and association, and smart contract of the agricultural product industry chain, to realize the openness, transparency, and seamless connection of information in all links of the industry chain.

**Figure 1:**
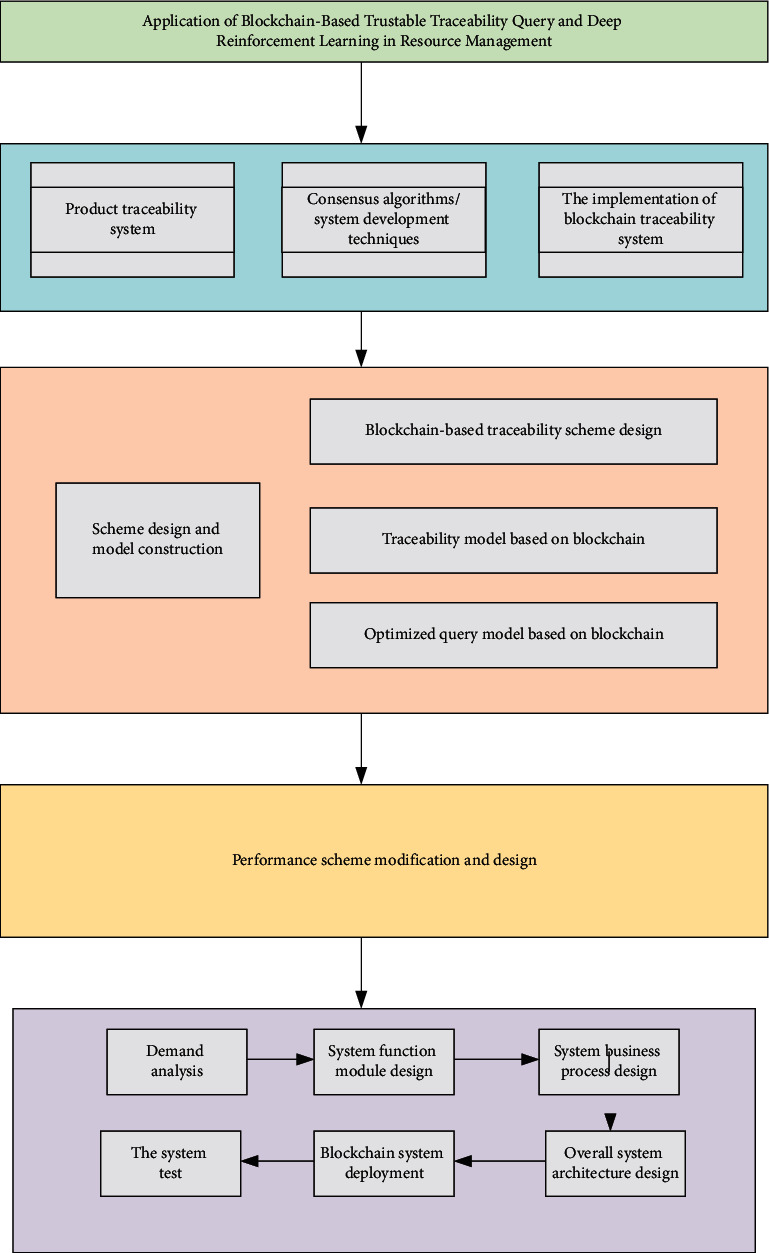
The technical framework.

Figure 1 presents the technical framework of the research scheme.

Table 1 lists the parameters and acronyms of the present work.

Innovatively, based on the BT, the traceability features of agricultural products, and the characteristics of the alliance chain, an analysis is made on the shortcomings of the consensus algorithm used in the consensus layer in the blockchain and improves the security of the blockchain network and the efficiency of block generation by adding the integral punishment mechanism. In addition, the Practical Byzantine Fault Tolerance (PBFT) consensus algorithm is improved, the relevant data of the flow process in the blockchain network are stored through the smart contract, and then the improved consensus algorithm is implemented on the platform.

## 2. Literature Review

Table 2 presents the cited literature.

Blockchain is the missing link of building a truly decentralized, untrusted, and secure environment of the IoT. Through the distributed network, advantages such as untempering and traceability of blockchain can help to provide a medium for the safe application of the IoT. “Blockchain + IoT” realizes the efficient transmission of credit between things without risk and leverage; then, it realizes the integration of capital flow, logistics, and information flow on the chain. Based on the interconnection of all things in the IoT, it ensures the credibility of all things, realizes the mapping of the physical world and the digital world, ensures the authenticity and integrity of the information on the chain, and further promotes the traceability development of the smart supply chain. Blockchain itself integrates distributed technology, cryptography, network security, and other technologies. In practical applications, there are still many problems leading to the blockchain system not running normally. Moreover, the distributed deployment of blockchain means that it needs to take up a huge amount of data storage, and decentralized distributed network communication can make communication efficiency worse. Blockchain technology has gone through three major stages of development, namely, the era of blockchain 1.0 with digital programmable currencies such as transfer and payment, the era of blockchain 2.0 integrating intelligent contract and blockchain technology, and the era of blockchain 3.0, when the confirmation, recording, and storage of all information bytes are realized on the Internet. At present, the development of blockchain technology is at stage 3.0. Blockchain technology has begun to be combined with industrial applications. Both in finance and traceability, blockchain technologies are constantly developing, which truly realizes the comprehensive promotion of blockchain applications. In the research of blockchain traceability, China mainly focuses on the immutability and openness of blockchain technology to achieve comprehensive traceability of data and uses advanced encryption technology to ensure data security, to realize an open, public, and credible traceability system. The traceability system of agricultural products supply chain based on alliance chain obtains the key data of agricultural products in the process of production, processing, logistics, and sales by using a variety of IoT acquisition and preservation methods. Simultaneously, it uses intelligent contracts to automatically execute transaction terms. Based on asymmetric encryption and digital signature, it ensures the uniqueness and security of transaction data and provides privacy protection through multichannel transaction isolation. It also ensures the reliable flow of information, capital, logistics, and business. In the agricultural products supply chain with a high degree of dispersion, long-chain, and multiple participants, it realizes the intelligent configuration of multiorganization efficient collaboration and resource consensus sharing and co-governance, which greatly reduces the cost of agricultural products supply chain. Blockchain technology involves networking, data collaboration, consensus algorithm, intelligent contract, privacy protection, and pattern standards. In the traceability technology of blockchain and the IoT, when the IoT is equipped with Radio Frequency Identification (RFID), RFID tags are often added to the products in the whole process of production, processing, logistics, and sales to save product information. The product information ciphertext is stored in the alliance chain for data verification and the use of RFID tags to record food identifiers. The unique identifier of food in the region is read through wireless communication equipment and sent to the data center for chain processing. The IoT equipment Lora is often used to reduce manual intervention data. Blockchain validation stores data. Intelligent contract script realizes automatic alarm, a credible, self-organizing, and open and transparent intelligent agricultural product traceability system. A sensor of the IoT is often used to record valuable information in the supply chain of agricultural products and directly put these data on the chain certificate.

The existing traceability system lacks unified upstream and downstream data definition standards and effective member collaboration mechanisms. When the traceability system involves complex industrial chains, the traceability data are often independent within a single independent participant system, which is difficult to open up. To obtain a complete traceability data link, there is often a need for much data collation work, and there is a high cost. Unified data format specifications and standard automated docking procedures help improve the efficiency of data tracking and coordination. In summary, the combination of blockchain technology and product traceability can well eliminate the disadvantages of traditional traceability. However, due to many links, long amplitude, and a large amount of data in most product industrial chains, blockchain systems often have problems such as low throughput, high consensus delay, and low query efficiency. Therefore, improving the consensus and query efficiency of blockchain traceability systems has always been a problem to be solved by scholars all over the world. The key to solving these problems is to establish a scientific blockchain traceability model to provide strong theoretical support for the improvement of the efficiency of the blockchain traceability system; on this basis, the blockchain consensus algorithm is further optimized to improve the efficiency of consensus and system performance.

## 3. BT and RM Technology

### 3.1. BT Analysis

**Figure 2:**
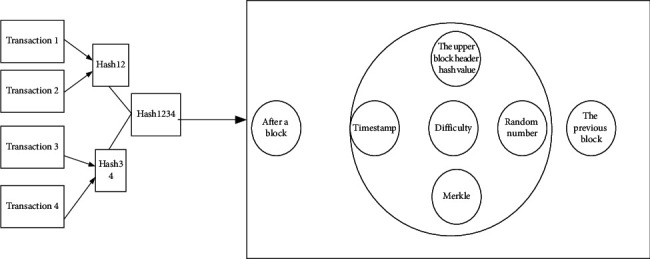
Scheme of a blockchain model.

Since 2008, BT has attracted growing attention as a solution to digital cryptocurrency transactions in untrusted distributed networks [12]. Blockchain is a set of data immutable records with timestamps, which are managed by independent computer clusters. Each of these data blocks uses encryption principles to protect and bind each other. A Blockchain network is a distributed network system without a central authority. There are many information encryption algorithms in blockchain for private node accounts, the most important of which is asymmetric encryption algorithm using public and private keys. Compared with the Internet technology, BT can transmit more information. To date, BT has seen various applications in many fields, such as evacuation behavior. Ostad-Ali-Askari designed a simulation of the subway station building evacuation using the Deep Neural Network (DNN) model [13]. BT is a relatively new and integrated computer network technology [14].  Figure 2 illustrates a simple blockchain model.

In Figure 2, the blockhead containing the previous Hash value is used to connect with the previous block. The timestamp records the block generation time. A 32-bit random number is generated by a block. Merkle is a 256-bit hash value based on all transactions in a block when accepting a transaction. The block body can weave the complete transaction information of the block into the Merkle tree, the construction process of which is to recursively calculate the hash value [15–17].

### 3.2. Analysis of the Data Traceability Model

**Figure 3:**
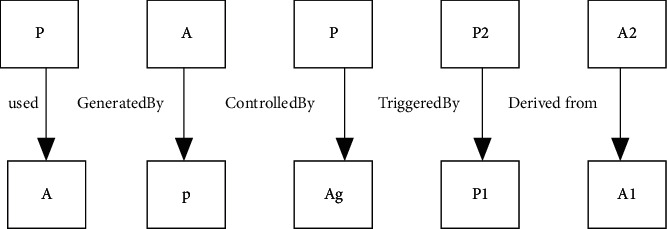
Scheme of OPM.

The main research contents of data traceability technology include three aspects of traceability description, traceability storage, and traceability application [18]. For data traceability, information collection is also very important. Lv et al. studied a monitoring system to collect real-time information [19]. The key and difficulty of data traceability technology lie in establishing an effective data model. There are two common data traceability models, namely, the Open Origin Model (OPM) and the Provenance (PROV) data model.  Figure 3 denotes the scheme of the OPM.

In the OPM, each traceability record is a Directed Acyclic Graph (DAG), where nodes include workpieces, processes, and agents. In Figure 2, *A* represents the workpiece, *P* presents the process, and Ag denotes the agent. The workpiece is an immutable state of things, which can be regarded as a physical object. Agents can promote, control, and affect the implementation of the process [20, 21].

Compared with the OPM, the simpler PROV data model is designed to transform traceability representations of domains or applications into generic models that can be swapped between systems. Lv and Xiu pointed out that the stability of mobile network systems was also related to the frequency of data signals [22]. PROV data model contains two-component types and relationships. Workpiece, activity, and agent belong to the type. The relationship between agents and activities is illustrated in the PROV data model. The attributes of workpieces in different versions of the PROV data model are not the same. BT is used to store traceability records of the PROV data model for two main reasons. One reason is that the PROV data model has strong scenario applicability. The other reason is that the PROV data model has relatively complete open-source codes [23, 24].

### 3.3. Design of a Trusted Traceability Survey on the Blockchain

**Figure 4:**
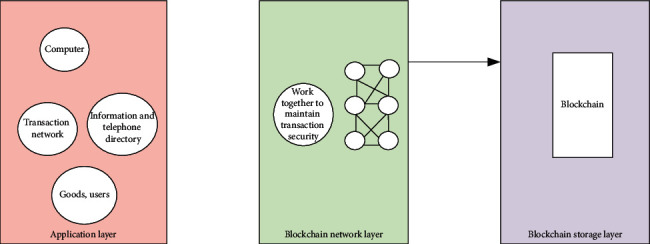
System architecture diagram.

Figure 4 represents the design of the proposed blockchain model framework.

According to Figure 4, the proposed blockchain model framework is divided into three layers from bottom to top: the storage layer, the network layer, and the application layer. The application layer consists of the transaction input module, consensus node display module, account information display module, and commodity traceability query module. The main functions of the network layer in the middle of the framework are to publish transaction information and block information. The storage layer at the bottom of the framework is responsible for storing the final block information after corroboration [25, 26].

**Figure 5:**
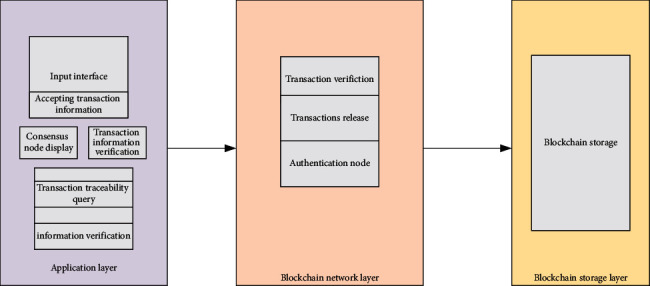
Design of the application layer.

The framework mainly improves the application layer and the network layer of blockchain. The application layer is used to interact with users. Besides, the blockchain network is built to form the network layer of the framework. Then, the transaction information is processed by the nodes participating in consensus in the blockchain network.  Figure 5 displays the design of the application layer.

In Figure 5, the application layer contains two parts of the access interface and the general function. The access interface is mainly used by the subject of the transaction to input the transaction information through the application layer, while the general module provides users with the traceability-related general functions. The internal structure of the blockchain network is the distributed deployment authentication node composed of all miner nodes. The miner node in the blockchain is responsible for finding out and verifying the transaction information and proof of work between transaction nodes [27, 28].

**Figure 6:**
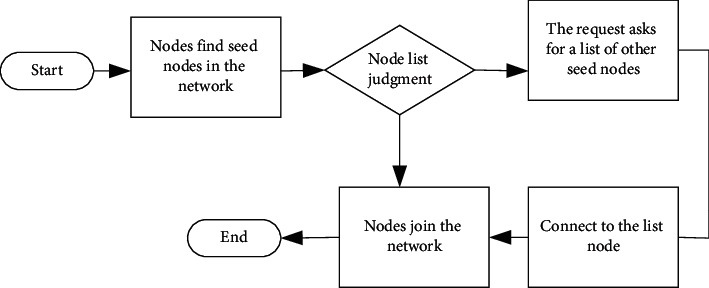
Flow chart of blockchain construction.

In the framework, the transaction roles on the whole blockchain can be considered as the existence of miner authentication nodes. At first, these nodes become consensus nodes through system application and connect with nodes with consensus behavior in the network. Then, they jointly form a network at the bottom of the blockchain, and finally, simulate to form a blockchain network.  Figure 6 signifies the process of node building a blockchain network [29]. The most important three steps are to establish connections with seed nodes, to get the address list of node links, and to connect to the nodes in the list. Zhang et al. proposed an agent-based modeling method to model multiagent environments and studied intelligent algorithms [30]. These ideas inspire experimental designs.

**Figure 7:**
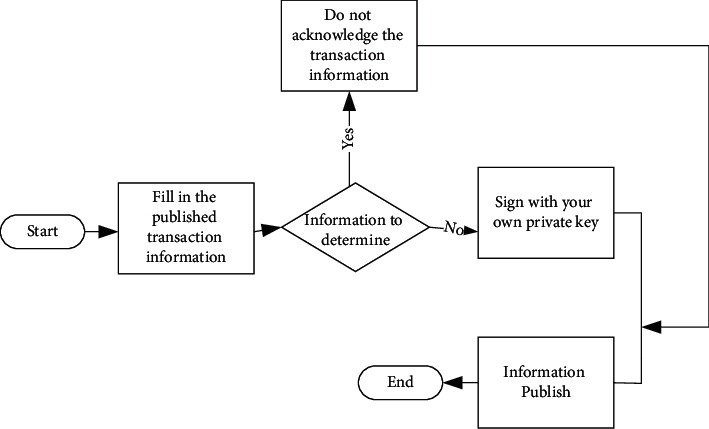
Transaction process between nodes.

After a node joins the network, there is an initial block locally. To connect with other nodes, the node is initialized by downloading data on the blockchain; meanwhile, to synchronize numerous blocks, the node also needs initialization [31, 32]. Proof of Work (POW) is introduced as the consensus mechanism with the advantages of easy implementation, good operation efficiency, and indestructibility. The focus of this model is the deployment system in the blockchain network. In the blockchain, after the consensus operation of nodes, if the transaction data are received by the block, all the participating transaction nodes will store the transaction content.  Figure 7 shows the transaction process between each node.

The transaction process involves three key steps. The first step is to fill in the transaction information by the consignee. The second step is to verify the filled information by logistics institutions. If the information is correct, the private key is used to sign the information. Otherwise, the information is discarded. Finally, the transaction is released to the network [33–35].

**Figure 8:**
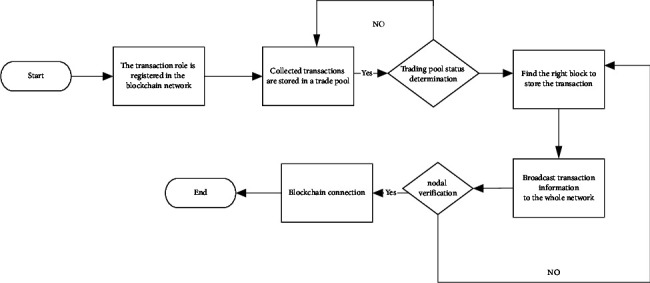
Transaction process between transaction roles.

Figure 8 illustrates the basic transaction process between trading roles.

Figure 8 indicates that the transaction process between transaction roles contains nine steps: (1) account registration; (2) transaction roles trade in the blockchain network; (3) transaction storage; (4) block searching; (5) searching of POF of transaction information by miner nodes; (6) whole network broadcast; (7) identification of the block-effectiveness by nodes; (8) blockchain extension; and (9) trade between retailers and customers through the network platform [16, 36].

**Figure 9:**
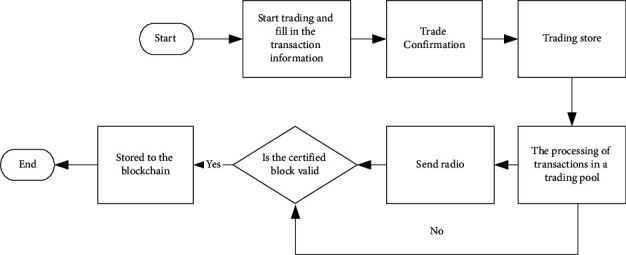
The implementation process of trade.

Figure 9 presents the implementation process of trade.

In Figure 9, the implementation process of trade contains five steps: (1) trading on the normal network and filling in the transaction information; (2) confirmation of goods receipt and spread of the transaction to the blockchain network; (3) combination of multiple transactions into a transaction group; (4) processing of transaction group by miner nodes; and (5) identification of the transaction effectiveness.

In the model, the technical principle of product traceability by customers is that after the successful transaction of consumers, the product can be traced on the blockchain through the product number. The system traces the previous block through the hash value contained in the current block and finds the initial transaction information of the product with the corresponding transaction number to achieve product source traceability. The specific process of source traceability is divided into two steps. The first step is to trace the historical transaction. The consigner first fills in and stores the transaction information. Then, the product traceability can be performed according to the blockchain structure and transaction number. The second step is that the traceability user can locate the position of the current transaction by inputting the transaction number through the client, and using this location to query the previous transaction of the current transaction.

### 3.4. BT-Based Traceability Scheme Design of Agricultural Products

**Figure 10:**
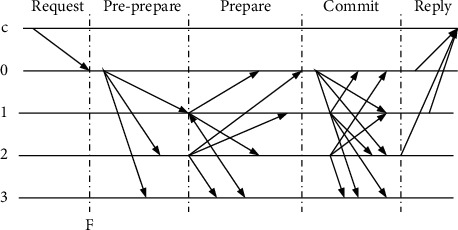
PBFT algorithm transfer diagram.

The consensus algorithm is the core of the whole blockchain. Generally, the trust problem of centralization needs to be effectively solved through the consensus mechanism. In a centralized network, each node is unreliable, which is not connected with one another, and cannot identify whether other nodes have betrayal or downtime, so they have to take huge efforts to ensure high information security, reliability, and accuracy. PBFT algorithm can maintain good consistency between distributed systems and Byzantine fault nodes; its advantages are low energy consumption and high efficiency.  Figure 10 displays the transmission diagram of the PBFT algorithm.

In Figure 10, *C* represents the Request node, lines 0, 1, and 2 all stand for the normal running servers, and line 3 represents the failed servers. The algorithm implementation is as follows: (1) Request: node C sends a request to the master node, which is recorded as 0. (2) Pre-prepare: after *C* requests the primary node server 0, the server 0 is passed to the secondary nodes 1, 2, and 3. (3) Prepare: after secondary nodes 1, 2, and 3 receive the delivery record, 1 continues to deliver to 023, 2 to 013, and 3 cannot continue to deliver because of failure. (4) Commit: if nodes 0, 1, 2, and 3 receive more than a specific number of the same requests in the Prepare phase, they will enter the Commit phase and pass the Commit applications. (5) Reply: for the Commit phase, if nodes 0, 1, 2, and 3 receive the same request in excess, the information will be fed back to node C. When the PBFT algorithm is directly applied to the alliance chain network of agricultural product traceability, there are some defects, such as poor control of failure nodes, and overload of network performance. Optimization is carried out on the PBFT algorithm to be more suitable for agricultural product traceability systems. The existing problems of PBFT are analyzed, and the integral penalty mechanism is used to improve the PBFT algorithm by forming a verification node list *L* through a part of trusted nodes and the nodes are given initial integral *IV*_*i*_=1. Each node needs to serve other nodes to maintain the integral. In each round of consensus, the best block is selected to package the verification nodes. The coefficient *λ* is used to reduce the integration of the worst block packaging verification node, namely, *IV*_*i*_=*λIV*_*i*_, *λ* ∈ (0,1). When the node integration in the verification node list *L* is lower than a specified value *ε*, the node will be cleared out of the list. When the remaining nodes in the list are less than 2/3, the list will be dissolved, and a new verification node column *L* will be regenerated. The improved PBFT algorithm can dynamically distribute verification power by adding the integral penalty mechanism and penalizing the failed nodes to improve the blockchain network security and block generation efficiency. The PBFT consensus algorithm is implemented through the selected verification nodes to avoid the participation of all nodes, thereby solving the high-bandwidth requirement of the PBFT and improving the dynamics of the consensus network.

### 3.5. DRL and RM

Network RM is the process of managing and distributing network process resources. In computer systems, RM refers to the technology used to manage computer resources. The advantage of network RM is that it can prevent the leakage of resources and deal with the requisition of resources. Real-time decision-making tasks in multi-RMS and RM networks are strongly dependent on the load and understanding of the environment. Recently, DL algorithms have been proposed to solve real-time decision-making problems. Nowadays, Reinforcement Learning (RL) has become an important branch of DL and Machine Learning (ML). RL makes continuously improved decisions using the agent to learn directly from the interaction with the environment. Therefore, RL is adopted to study the task scheduling problem of resource clusters in cloud computing RM in the experiment. Cloud computing manages various virtualized resources, where scheduling becomes a key part. The main idea of task scheduling is to reasonably adjust tasks to minimize time loss and better utilize resources.

In the context of cloud computing, RM is the process of allocating computing, storage, network, and energy resources to a group of applications by seeking to jointly meet the performance objectives of applications, namely, infrastructure providers and cloud resource users. The goal of the provider is to use resources efficiently and effectively under the constraints of service-level agreements with cloud users. The effective utilization of resources is usually realized by virtualization technology, which promotes the statistical reuse of resources across customers and applications. The goals of cloud users usually focus on application performance, availability, and cost-effective expansion of available resources according to changing application requirements. Typically, these goals are accompanied by resource-specific constraints to meet nonfunctional requirements related to security or legitimacy. The cloud environment RMS can be divided into eight modules according to its functions. The task scheduling module studied here belongs to the global scheduling module of virtual resources in the cloud environment RMS.

**Figure 11:**
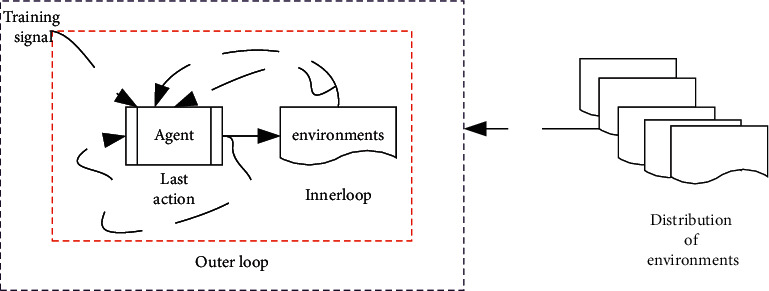
Scheme of RL.

Interactive learning is the connotation of RL, the essence of which is the interaction between RL and environments, in the form of changing its own behavior by observing the other's behavior. Optimal control is another key influence on RL. The autonomous agent controlled by the machine learning algorithm observes its environment state at time step *t* and interacts with the environment by taking actions in the state; it is shown in Figure 11. When the agent acts, the environment and the agent convert to a new state according to the current state and the selected action.

DL technology partly improves object detection, speech recognition, and language translation technology. The foremost feature of DL is to automatically find the low-dimensional representation of high-dimensional data. DL also accelerates the progress of RL, enabling RL to expand to complex decision problems that are difficult to deal with before. There are two methods to optimize RL, which are based on value function and strategy search. There is also a mixed actor-critic method, which uses both value function and strategy search. At present, only the method based on value function, namely Q-learning, is adopted. Equation (1) illustrates the algorithm updated by Q-learning.

(1) $$\begin{array}{c} Q\left(s_{t},a_{t}\right)\leftarrow Q\left(s_{t},a_{t}\right)+\alpha \left[R_{t+1}+\gamma \max_{\alpha }Q\left(s_{t+1},a\right)-Q\left(s_{t},a_{t}\right)\right]\text{.} \end{array}$$

In (1), *α* is the amplitude of update, while *γ* is the discount factor (attenuation).

Q Learning-Convolutional Neural Network (DQN) is an algorithm using experience playback technology. In the internal loop of the algorithm, Q-learning upgrades and updates a small batch of the experience samples randomly selected from the sample storage pool. After performing the experience playback, the agent selects and acts according to the *ε*-grey strategy. Since it is impossible to use any length of a neural network to input, a fixed length of the input is generated by the function *φ*. The algorithm design is listed orderly according to the basic elements of RL as follows. (1) State-space: using the remaining computer resources in the cluster (task scheduling should be consistent with the current remaining resources to reach the resource requirements of the task; the waiting time of the task affects the optimization goal), each priority of all tasks represents the system state. (2) Action space: in each time slot, the action performed by the agent belongs to any subset of *M* tasks. Many forms of function approximations can represent *Q* function, such as the linear combination of state/action space features, decision tree, and nearest neighbor function. As mentioned earlier, DNN has recently been successfully used as a function approximation to solve large-scale RL tasks. One advantage of DNN is that it finds features automatically, so it is used in the design to represent the *Q* function. Taking the current observed system state as input, the *Q* value of each action (the current *M* optional actions) is output, and the action with the maximum *Q* value is selected as the final task. The DNN is trained by the iterative method with a fixed number of tasks arriving in each iteration. When all tasks are completed, the iteration ends. To train the generality of the algorithm, *M* different tasks are trained for the arrival sequence, and each sequence is called a task set. In each iteration, each task set is trained *N* times, and the *Q* table (the experience sample storage area) generated by the training of all task sets is stored together. After each fixed interval time slot, the experience sample is extracted from the *Q* table to learn.

**Figure 12:**
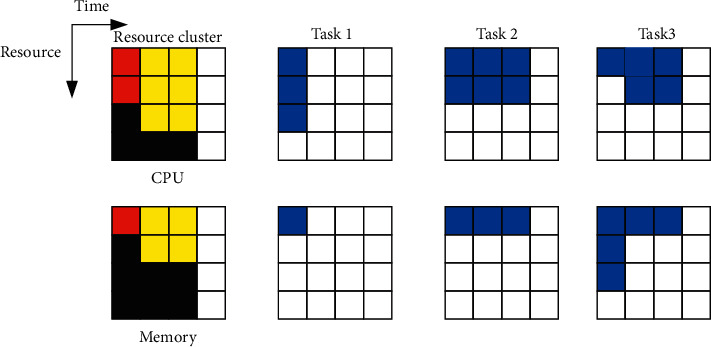
State diagram.

The task scheduling algorithm combined with DQN is designed as follows: the remaining computer resources in the cluster are used to reach the resource demand of the task, the waiting time of the waiting task, and the priority of all tasks represent the state of the system.  Figure 12 shows a visualization of the resource requirements of the task and the allocation status of the cluster resources.

**Figure 13:**
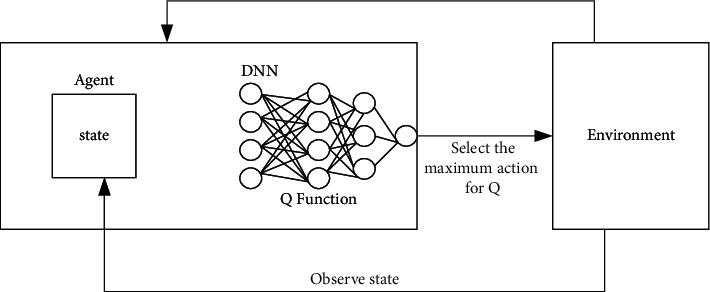
Structure diagram of RL network using DNN.

The cluster image in Figure 12 indicates that each resource is allocated to the scheduled tasks. The resource allocation status of *T* time slots in the future is presented from the current time slot all the way to the left. Different colors in the image represent different tasks: red and yellow represent single-stage tasks, and black represents multistage tasks. The image on the right side of Figure 12 represents the resource requirements of waiting tasks, which also include multistage tasks. In an ideal state, users consider all waiting tasks in the current system. However, for the neural network to have fixed input, only the first *M* arriving tasks are considered. Information about any task other than the first *M* is in backlog, and the backlog records all the information of the task. In this way, the algorithm focuses on the tasks that arrive earlier, making the learning process more efficient. Many forms of functions can be regarded as the *Q* function in the DQN algorithm. During the proposed algorithm design, the neural network is used to represent the *Q* function, which does not need to find features artificially.  Figure 13 denotes the schematic diagram of an RL DQN algorithm using DNN.

**Figure 14:**
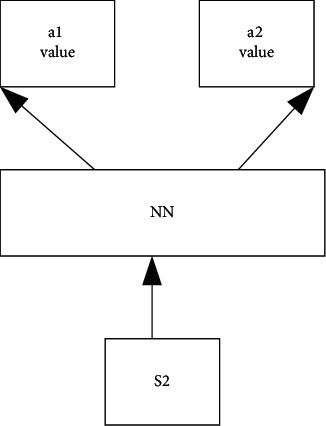
Neural network output.

Setting of the reward signal is to help the agent learn the strategy suitable for the optimization goal. The reward for minimizing the average task slowdown of the task is ∑_*j*∈*J*_ − 1/*T*_*j*_, where *J* means the collection of current waiting tasks, and the cumulative reward is consistent with the negative value of the sum of task slowdown over time. Therefore, within the scope of this paper, maximizing the reward is equivalent to minimizing the average task slowdown. The neural network function is used to approximate the *Q* function, the currently observed system state is taken as input, each action is output, and the action with the largest *Q* value is selected as the final execution task. The neural network is trained by the iterative method. For each iteration, a fixed number of tasks arrive. When all tasks are completed, this iteration ends. In each iteration, each task set is trained *N* times, and the *Q* table generated by the training of all task sets is stored together. After every fixed interval of time slot, empirical samples are extracted from the *Q* table for learning. Consistent with the previous neural network, the neural network needs the training to predict the accurate value. However, in RL, the training method of the neural network is different from supervised learning. The learning goal of supervised learning before learning is fixed. If the input state of the neural network is s_2_, then the *Q* value of output actions *a*_1_ is shown in Figure 14.

Figure 14 illustrates the output of the neural network. At first, the correct *Q* values of *a*1 and *a*2 are required, and a *Q* prediction is also required to update the neural network. Thus, the parameters of the neural network = (old neural network parameters + learning rate *α*) *∗* (real *Q* − predicted *Q*).

Objective function is a quantifiable feedback signal provided by the environment to the agent. Its main purpose is to evaluate the performance of the agent in its current state. In the beginning, resource scheduling objectives for the current data center need to be specified. Two objective functions are set here. First, the average weighted turnover time should be minimized. Second, minimization needs to be realized on the average turnaround time. From the point of view of a job, the most concerning result is how long the operation of the current job takes, and the main assessment indicators are the average turnover time and the average weighted turnover time.

### 3.6. Experimental Environment Configuration and Experimental Methods

All the experiments are completed under the environment of the Tencent Cloud platform. The Apache Tomcat software is used in the Web front end, and the traceability data are stored in the MySQL database before access.  Table 3 illustrates the environmental configuration.

The experiment is deployed with the Golang environment, Docker, and Fabric. The program of Golang can be obtained and installed from its official website. The ect/profile file needs to be modified to adapt to all users because the link code is used for programming by Golang and multiple participants in the system use the link code to trade. A complete Docker includes a client, a daemon, a mirror, and a container. It is necessary to configure the application for defining and running multiple containers in docker-compose. When the source code of Hyperledger Fabric hosted in Github is used, it is necessary to obtain the image files needed to run Fabric and deploy them locally, including fabric tools, fabric-peer, fabric-CouchDB, and fabric-ca. Finally, the relevant environmental variables are configured. In the storage overhead test, the size of the trigger window of each processing unit is set to 1, and the number of processing units varies from 1 to 10. Meanwhile, the data tuple size is 200 B and 2 K, and the state snapshots of the processing unit are 200 B and 2 K, respectively. Moreover, there are three groups of time overhead tests, with the unit delay of the processing unit of 0 millisecond, 1 millisecond, 2 milliseconds, and 3 milliseconds.

The initial setting of task load in the task scheduling evaluation method of computing cluster in DRL cloud computing is as follows. For the pure phase task set, 80% of the task length is 1 t–2 t of random uniform distribution, and the remaining task length is 15 t–18 t of random uniform distribution. Assuming that the system has a major resource demand and a secondary resource demand, and the priority of the task has two levels and random distribution, multistage tasks can be divided into two stages accordingly. The task length of the first stage is 15 t–18 t of random uniform distribution, and the task of the second stage is 1 t–2 t of random uniform distribution. The mixed set with single-stage and multistage tasks contains 50% single-stage tasks and 50% multistage tasks. Single-stage and multistage tasks arrive in Poisson distribution, and the task arrival rate determines the change of task load rate. General performance business indexes are the average task slowdown and the average task completion time of the task set.

## 4. Traceability Experiment Using Blockchain

### 4.1. Analysis of Field Test Results of Traceability Using Blockchain

**Figure 15:**
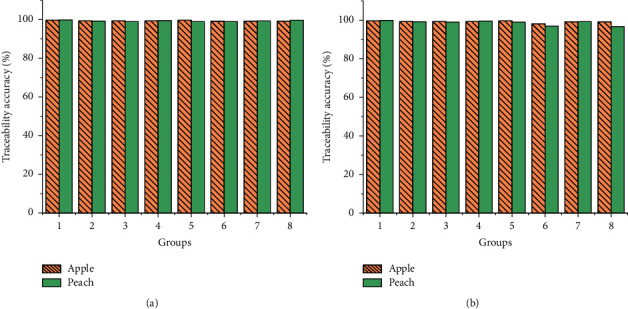
Results of the traceability test. (a) The first test; (b) the second test.

Figure 15 presents the traceability test results of 100 peaches and apples in 8 groups.

Figure 15 reveals that the traceability accuracy of the system constructed here is 99% in the first group of samples. The traceability accuracy of the constructed system is 98% in the second group. Overall, the traceability accuracy is above 98% for the proposed system in 8 groups of experimental samples, and the traceability accuracy is close in each experimental group. The traceability accuracy of peach is slightly higher than that of apple. The reason may be that the origin of apple is more than that of peach.

### 4.2. Comparison on the Optimization Objects

**Figure 16:**
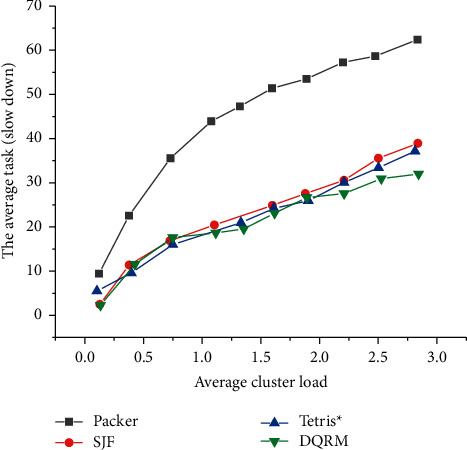
Comparison of average task slowdown of different algorithms under different load levels under single-stage task.

Figure 16 illustrates the comparison between the performance in the task slowdown of Deep D-Networks Resource Management (DQRM) with that of other algorithms at different load levels, when the task set is a single-stage task.

As expected, experimental results reveal that the average task slowdown increases with cluster load, and Shortest Job First (SJF) outperforms Packer, Tetris^*∗*^. Figure 16 also demonstrates that DQRM has better performance than all heuristic methods. As the load increases, DQRM outperforms Tetris^*∗*^ at higher loads because it automatically learns to retain some resources; while in Tetris^*∗*^, small-length tasks may sometimes wait for the existing large-length tasks to be completed, because Tetris^*∗*^ is a task-protective algorithm.

**Figure 17:**
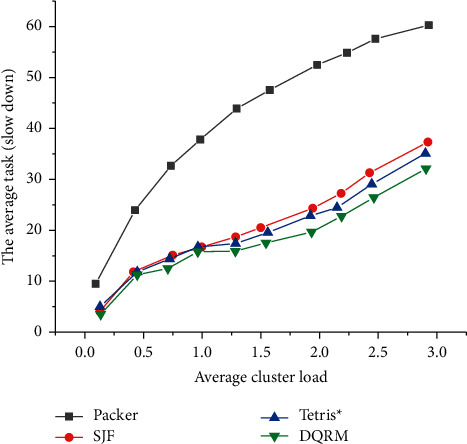
The average task slowdown under different load levels in the mixed phase task set.

Figure 17 displays the comparison of the average task slowdown of different algorithms under different load levels under mixed tasks.

It is clearly illustrated in Figure 17 that in the mixed task set composed of single-stage and multistage mixed tasks, the trend of the task length distribution is generally consistent with that of the single-stage task length distribution. Therefore, it can be explained that the strategy that the proposed algorithm needs to learn in the single-stage and multistage mixed tasks is still the reservation of resources, so that the tasks with smaller length can be quickly arranged. This also proves that the proposed algorithm has a certain meaning and can solve simple multistage tasks.

**Figure 18:**
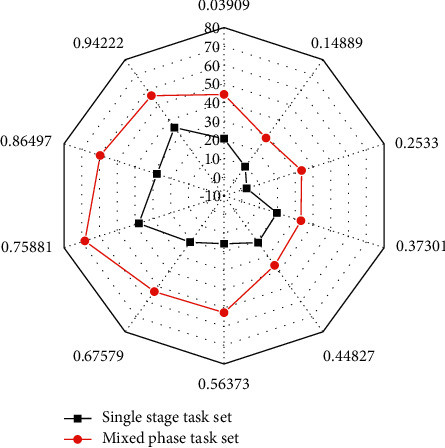
Comparison of iterations between two task sets at different task arrival rates.

Since the load of single-stage and mixed-stage task sets is determined by the task arrival rate, the task load of each task is not the same under the same task arrival rate.  Figure 18 demonstrates the comparison of the number of iterations of the two task sets under different task arrival rates.

Overall, DQRM actually learns the same strategy, but the task becomes more complex, that is, there are more changes in task conversion to state representation. For systems, larger state space requires more iterations to learn.

**Figure 19:**
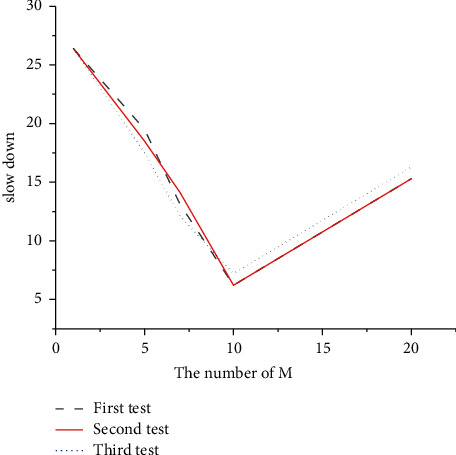
Average task slowdown for top *m* tasks.

Figure 19 bespeaks the results of DQRM algorithm after 100 iterations under the condition of different *M* parameter settings and other initial parameters unchanged, and the results when the task load of single-stage task set is 1.5.

Figure 19 indicates that reducing *M* is conductive to reducing the action space and reducing the difficulty of DQRM learning, but there appears a smaller number of waiting tasks considered by the system at each step and a smaller number of the strategy space. When the optimization goal is the average task slowdown, to reduce the task slowdown of all tasks, when the optional tasks become fewer and fewer, the tasks can only be executed according to the task arrival order randomly generated by the task set, and there is no optimization space. Consequently, DQRM cannot effectively reduce the average task slowdown. When *M* is greatly increased, the action space is increased, and the difficulty of DQRM learning is increased. In the experiment, when *M* is 20, the iteration time of each training is about four times that when *M* is 10, which is not conducive to rapid convergence. And actually, the number of fixed task arrival is not suitable for large M, because it will make the system observed state value very sparse, nor conducive to training learning. When *M* is 8, although the training time per iteration is the shortest, the improvement over Tetris^*∗*^ on the optimization objective is not optimal. When *M* is 11, although the performance is improved, the time taken for one iteration is too high. Because *M* is 10, consideration is only given to the first 10 arriving tasks, which is indeed a relatively optimal choice.

**Figure 20:**
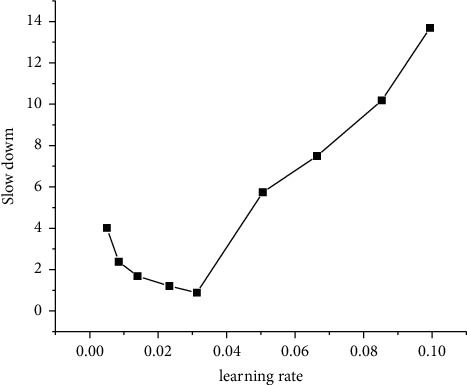
Average task slowdown under different learning rates.

Figure 20 displays the changes in algorithm performance when the learning rates are 0.1, 0.03, 0.01, 0.003, and 0.001, respectively, with other parameters unchanged.

Figure 20 implies that the average task slowdown of the algorithm is not better than that of Tetris^*∗*^ when the learning rate is 0.003 or below, because the overfitting occurs when the learning rate is low and the direction of the decline cannot be quickly found, which requires more learning time. When the learning rate increases, the final value of average task slowdown increases sharply. Because of the large learning rate, the neural network is more inclined to learn the characteristics of the order of arrival and resource requirements of the current batch of tasks at each update. However, in the process of reinforcement learning, through continuous iteration, the learning samples generated by the system are increasing, and there will be a gradual increase in the diversity of the samples. The samples will cover more possible optimal task scheduling order. Therefore, the higher learning rate will make the feedback reward, it resulted in the average task slowdown easy to fall into local optimum.

**Figure 21:**
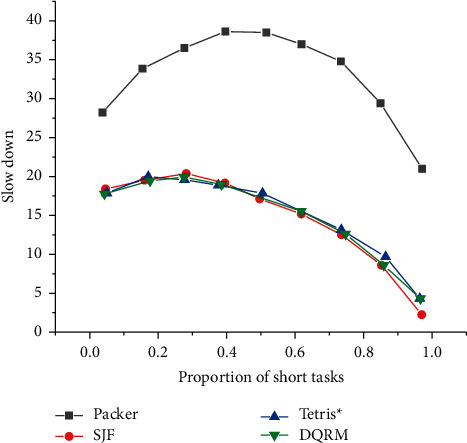
Average task slowdown under different short task proportions.

Figure 21 presents the comparison between DQRM with other algorithms when the proportion of short tasks is 10% to 90% and the task arrival rate is 0.7.

Figure 21 indicates that when the proportion of short tasks changes from 10% to 50%, the DQRM algorithm has no advantage. When the proportion of short tasks is gradually greater than that of long tasks, the DQRM algorithm gradually learns a better strategy. Therefore, when the proportion of long tasks in the task environment is only 10%, the DQRM algorithm has the largest proportion of improvement compared with Tetris^*∗*^.

### 4.3. Comparison among Traceability Algorithms

Under the single-stage task set load, Table 4 demonstrates the performance of all algorithms for the two optimization objectives (average task slowdown and average task completion time) when the cluster is under high load (load = 1.50).

Table 4 displays that Tetris^*∗*^ is superior to other heuristic methods. Yet, after Deep D-Networks Resource Management (DQRM) learns the strategy to optimize the goal through appropriate rewards, each index can get the best performance. Therefore, DQRM can learn specific strategies according to different optimization objectives.

Table 5 shows the performance of all algorithms for the two optimization objectives (average task slowdown and average task completion time) under the mixed-phase task set loads and high cluster load (load = 1.5).

Table 5 presents that DQRM can still learn the strategy to optimize the goal through appropriate rewards and can get the best performance for each index. Therefore, even under different mixed-phase task loads, DQRM can learn specific strategies according to different optimization objectives. DQRM has been proved to be extensible.

**Figure 22:**
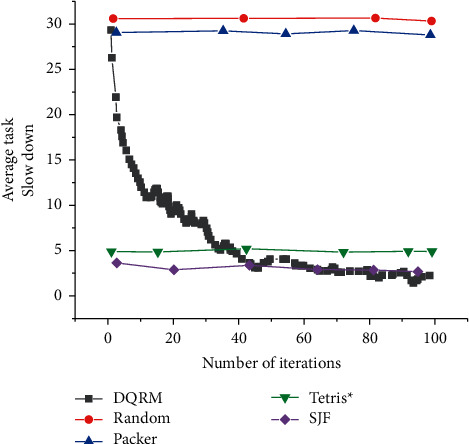
Learning curve.

**Figure 23:**
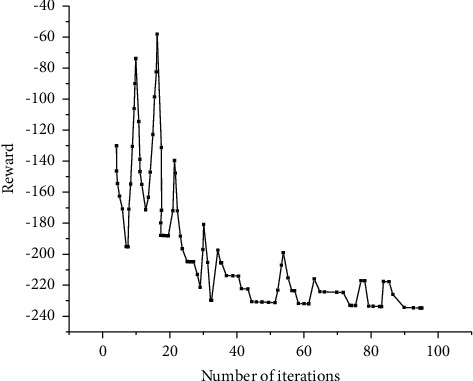
Reward curve.

Figure 22 indicates the average task slowdown index of the DQRM algorithm, random algorithm, packer algorithm, Tetris^*∗*^ algorithm, and SJF algorithm at the end of each iteration, and Figure 23 signifies the reward after each iteration.

Figures 22 and 23 suggest that the DQRM algorithm only needs 100 iterations to perform as well as Tetris^*∗*^ algorithm. Thus, the DQRM algorithm has a faster convergence speed over Tetris^*∗*^ algorithm. This is because the DQRM algorithm represents the task resource requirements as the image state and directly serves as the input of the neural network, so the network training speed is faster. The reward curve in Figure 23 implies that the reward increases with the increase of algorithm iteration times.

### 4.4. Display of Main Functions of the Agricultural Product Traceability System

**Figure 24:**
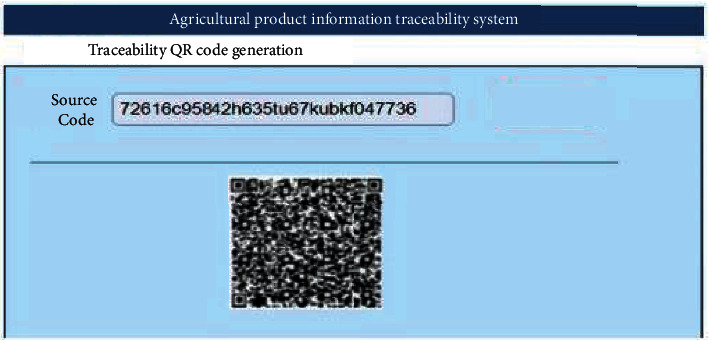
Traceability QR code generation interface.

The front end of the agricultural products traceability system, the World Wide Web (Web), mainly uses Hypertext Markup Language 5 (HTML5) and Cascading Style sheets (CSS) to deploy operations on dream weaver, and the back end uses MySQL database to read data. The data are collected in the field.  Figure 24 denotes the traceability-enabled QR code generation interface.

**Figure 25:**
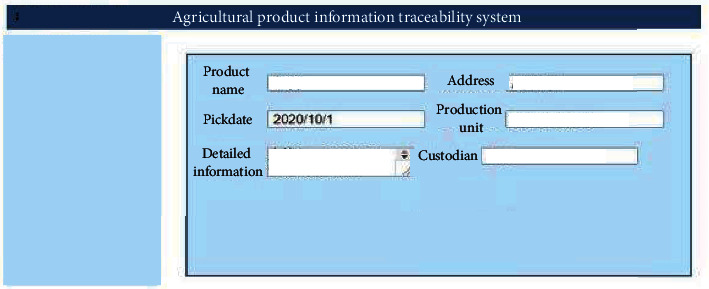
Information release interface for the agricultural products.

Figure 25 presents the information release interface for the agricultural products.

**Figure 26:**
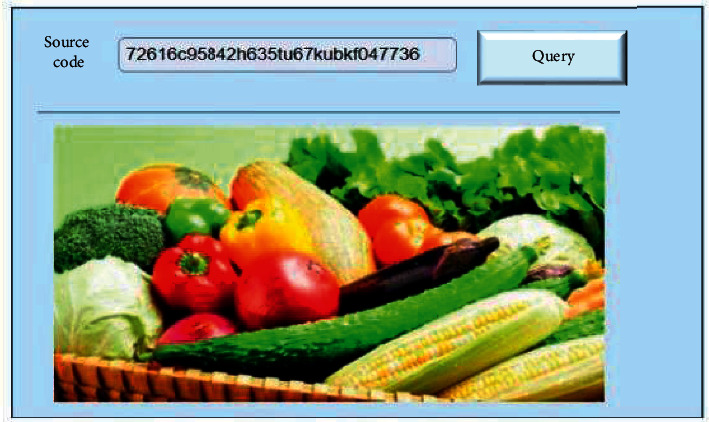
Traceability code query interface.

After buying agricultural products, consumers can query the production, sales, and transportation information in detail through different ways, such as QR code scanning and traceability code input.  Figure 26 shows the query interface for inputting agricultural product traceability code.

Comparing the proposed traceability system with the traceability system constructed by Yang et al. [37], the advantage of the proposed system is that the system solves the problems of asymmetric information, difficult sharing, easy tampering, and centralized storage in the IoT-based agricultural products traceability system of previous researchers, thus making the whole chain of agricultural product information credible and reliable [38–42]. Meanwhile, the proposed system also overcomes the common problems of information storage isolation, data sharing of information systems, and difficult interaction in the traceability of traditional agricultural products [43–47].

## 5. Conclusion

The focus of the present work is to study the trusted traceability problem and resource management problem from the perspective of blockchain and edge computing. The present work takes the resource management problem in computer system and network as the starting point, and understands the difficulties of accurate modeling of existing heuristic methods in complex environments and poor scalability in different environments. Through in-depth understanding of the characteristics of deep reinforcement learning, efforts are made to apply the proposed algorithm to improve the storage performance of blockchain in different network scenarios. For the task scheduling problem of resource clusters in cloud computing resource management, the present work refers to the existing multiresource demand task scheduling model, expands the task type model, and uses the DQN algorithm in deep reinforcement learning to solve various optimization objectives preset in the task scheduling problem. Primarily, the task model is extended to single-phase task and multiphase task, and then the task has priority. Next, the deep reinforcement learning algorithm is used to learn the strategies of complex optimization objectives including minimizing the average task slowdown, minimizing the average task completion time, and maximizing the task priority discrimination. Finally, the performance of the algorithm is analyzed from various parameters and structural levels of the algorithm and task load. Aiming at the problems existing in the traditional traceability system of agricultural products, the present work designs the traceability scheme of block chain, such as process, data storage and association, intelligent contract, and realizes the open, transparent, and seamless connection of information in each link of the industrial chain. However, there are still some shortcomings. In the experiment, more task modes are not considered, such as not simulating the common inter-task dependencies in parallel tasks. In addition, the resource requirements of the task may not be announced in advance, and the scheduling program may only obtain accurate resource requirements when the task is running. In the next step, related researches will be conducted on complex task models and inaccurate task resource requirements.

## Figures and Tables

**Table 1:** Acronym.

Acronym	Explication	Abbreviation	Explication
NFC	Near field communication	PROV	Provenance
GSM	Global system for mobile communications	ALL	Reinforcement learning
DES	Data encryption standard	*α*	Amplitude of update
RSA	Rivest–Shamir–Adleman	*γ*	Discount factor (attenuation value)
QOS	Quality of service	DQN	Q learning-CNN
Device to device	D2D	Deep neural networks	DNNs
OPM	Open origin model		

**Table 2:** Literature review.

Classification of proposed approaches	Year	Authors	Strengths	Gaps	Objectives	Field of application	Constraints
Trusted traceability query of blockchain	2021	Vikaliana et al. [4]	It can quickly summarize and sort out the literature	High complexity	Helps the system to carry out the literature review	Traceability of agricultural enterprise commodities	The limited scope of use
	2019	Chen et al. [5]	Three main areas of enterprise management, user query, and government supervision are designed to track information flow and system	High cost	The application value hypothesis of NFC technology in the agricultural product supply chain is proposed and verified	Improvement of the agricultural product supply chain	The dataset used is small
	2019	George et al. [6]	In addition to enhancing the traceability of food (products), the prototype can grade the quality of food consumed by human beings	There are few actual use scenarios	A restaurant prototype using blockchain and product identification to achieve more reliable food traceability is proposed	Restaurants	The system construction is complex
	2021	Yang et al. [7]	Traceability of product information in product supply chain	It needs to be used with the database	It improves the transparency and credibility of traceability information	Agriculture products	The limited scope of use
	2021	Liu et al. [8]	Exact distance query	Logistics, transportation, and product traceability	It solves the problems of data leakage and query leakage in data outsourcing	Products	The performance requirements of computing equipment are high

RM	2021	Munaye et al. [9]	The scheme has good results. In the evaluation task, the evaluation results converge quickly, which is suitable for heterogeneous IoT (IoT) networks with low complexity	The experimental object is relatively single	The resource use of IoT networks is optimized	Wireless networks	The performance requirements of computing equipment are high
	2020	Chen et al. [10]	Each scheduling slot makes decentralized optimal band allocation and packet scheduling decisions. The performance of the previous algorithm is greatly improved	The experiment is carried out only under ideal conditions	Radio RM	Wireless networks	High performance requirements for computing equipment
	2020	Yang et al. [11]	The priority experience replay and coordinated learning mechanism are adopted to enable distributed communication links, which improve the network performance and access success probability	The algorithm is tested only under ideal conditions	The radio block joint allocation and transmission power control strategy are optimized	Wireless networks	High requirements for the hardware equipment

**Table 3:** Environment configuration.

Type	Name	Parameter
Hardware	Tencent Cloud	System: Centos 7.0; RAM: 2G; CPU: 1H; network card: 1M

Software	cURL	Version: 7.61.0
	Golang	Version: 1.11
	Docker	Version: 18.03.1-ce
	Docker-compose	Version: 1.14.2
	Fabric	Version: release-1.2
	Apache Tomcat	Version: 7.0.0

**Table 4:** Average task slowdown and average task completion time of single-stage task set.

Algorithm	DQRM	Tetris^*∗*^	SJF	Packer
Average task slowdown	6.1	7.2	8.8	32.5
Average task completion time	18.2	19.1	20.1	48.6

**Table 5:** Average task slowdown and average task completion time of mixed-phase task set.

Algorithm	DQRM	Tetris^*∗*^	SJF	Packer
Average task slowdown	5.1	5.8	6	26.1
Average task completion time	16.2	18.1	19.1	38.6

## Data Availability

The raw data supporting the conclusions of this article will be made available by the corresponding author, upon request.
